# Lupeol protect against LPS-induced neuroinflammation and amyloid beta in adult mouse hippocampus

**DOI:** 10.3389/fnut.2024.1414696

**Published:** 2024-07-10

**Authors:** Kyonghwan Choe, Jun Sung Park, Hyun Young Park, Muhammad Tahir, Tae Ju Park, Myeong Ok Kim

**Affiliations:** ^1^Division of Life Science and Applied Life Science (BK21 FOUR), College of Natural Sciences, Gyeongsang National University, Jinju-si, Republic of Korea; ^2^Department of Psychiatry and Neuropsychology, School for Mental Health and Neuroscience (MHeNs), Maastricht University, Maastricht, Netherlands; ^3^Department of Pediatrics, Maastricht University Medical Center (MUMC+), Maastricht, Netherlands; ^4^Haemato-Oncology/Systems Medicine Group, Paul O’Gorman Leukaemia Research Centre, Institute of Cancer Sciences, College of Medical, Veterinary and Life Sciences (MVLS), University of Glasgow, Glasgow, United Kingdom; ^5^Alz-Dementia Korea Co., Jinju, Republic of Korea

**Keywords:** Alzheimer’s disease, lipopolysaccharide, lupeol, amyloid-beta, neuroinflammation

## Abstract

Neuroinflammation includes the activation of immune glial cells in the central nervous system, release pro-inflammatory cytokines, which disrupt normal neural function and contribute to various neurological disorders, including Alzheimer’s disease (AD), Parkinson’s disease, multiple sclerosis, and stroke. AD is characterized by various factors including amyloidogenesis, synaptic dysfunction, memory impairment and neuroinflammation. Lipopolysaccharide (LPS) constitutes a vital element of membrane of the gram-negative bacterial cell, triggering vigorous neuroinflammation and facilitating neurodegeneration. Lupeol, a naturally occurring pentacyclic triterpene, has demonstrated several pharmacological properties, notably its anti-inflammatory activity. In this study, we evaluated the anti-inflammatory and anti-Alzheimer activity of lupeol in lipopolysaccharide (LPS)-injected mice model. LPS (250ug/kg) was administered intraperitoneally to C57BL/6 N male mice for 1 week to induce neuroinflammation and cognitive impairment. For biochemical analysis, acetylcholinesterase (AChE) assay, western blotting and confocal microscopy were performed. AChE, western blot and immunofluorescence results showed that lupeol treatment (50 mg/kg) along with LPS administration significantly inhibited the LPS-induced activation of neuroinflammatory mediators and cytokines like nuclear factor (NF-κB), tumor necrosis factor (TNF-α), cyclooxygenase (COX-2) and interleukin (IL-1β). Furthermore, we found that LPS-induced systemic inflammation lead to Alzheimer’s symptoms as LPS treatment enhances level of amyloid beta (Aβ), amyloid precursor protein (APP), Beta-site APP cleaving enzyme (BACE-1) and hyperphosphorylated Tau (p-Tau). Lupeol treatment reversed the LPS-induced elevated level of Aβ, APP, BACE-1 and p-Tau in the hippocampus, showing anti-Alzheimer’s properties. It is also determined that lupeol prevented LPS-induced synaptic dysfunction via enhanced expression of pre-and post-synaptic markers like SNAP-23, synaptophysin and PSD-95. Overall, our study shows that lupeol prevents memory impairment and synaptic dysfunction via inhibition of neuroinflammatory processes. Hence, we suggest that lupeol might be a useful therapeutic agent in prevention of neuroinflammation-induced neurological disorders like AD.

## 1 Introduction

Neuroinflammation involves the activation of immune system of the central nervous system (CNS), which includes glial cells like microglia and astrocytes, to protect against infections, injuries, or neurological diseases and maintain neuronal homeostasis (1, 2). Evidence suggests that neuroinflammation plays a key role in Alzheimer’s disease (AD) development. Studies have shown that systemic inflammation or septic shock can lead to memory impairment and neuronal loss in animals (3, 4). Lipopolysaccharide (LPS), an endotoxin from gram-negative bacteria, triggers immune responses and inflammation. Research indicates that systemic LPS administration increases the production of inflammatory mediators such as nitric oxide synthase (NOS-2), cyclooxygenase (COX-2), and cytokines like TNF-α, IL-1, and IL-6, causing various neurobiological effects (5, 6). The major ligand for LPS is the TLR4 receptor which activates the nuclear factor (NF-κB) downstream signaling pathway (7, 8). Progressive neuroinflammation has been shown to induce neurodegeneration in the different forms of brain disorders like sepsis, AD, Parkinson’s disease and multiple sclerosis, etc. (9–11). Although the exact mechanism for LPS to induce Alzheimer’s pathogenesis is not fully elucidated, various research studies described that LPS-induced neuroinflammation impairs memory function via increased accumulation of amyloid beta and inactivation of beta (β) and gamma (γ) secretases. Other studies have shown that inflammatory mediators or cytokines like NOS-2, Prostaglandins, IL-1β, IL-6, TNF-α or transforming growth factors (TGF) can induce augmented expression of amyloid precursor protein (APP) and generation of amyloid beta (Aβ) (12, 13). Furthermore, it has also been stated that the promoter region of β-secretase (BACE) has NFκB binding site which may regulate Aβ formation (14). Likewise, other studies showed that LPS administration impairs memory performance and induces synaptic dysfunction (15, 16).

Recently, there has been extensive research into the therapeutic potential of natural products sourced from plants, along with their bioactive components, in addressing various neurodegenerative diseases such as Alzheimer’s (AD), Huntington’s (HD), and Parkinson’s (PD) (17). Lupeol is a nutritional pentacyclic triterpene found in various medicinal plants and fruits like mango, olive, and strawberry. Lupeol exhibits a wide range of biological properties as experimental studies have shown that lupeol possess strong anti-oxidant, anti-inflammatory, anti-diabetic, anti-mutagenic, antineoplastic, and hepatoprotective properties (18, 19). Moreover, *in vitro* studies have described the neuroprotective effect of lupeol in different types of cells like mouse hippocampal HT22 cells, hepatocytes, and glioma cells. The anti-inflammatory and anti-oxidant activity of lupeol mainly contributed to its protective capability in hepatic or gastric disturbances (20, 21). In this study, we evaluated the effect of systemic administration of LPS on proinflammatory cytokines, Aβ production, Tau hyperphosphorylation, and proteins associated with synaptic dysfunction in the hippocampus. We determined the inhibitory role of lupeol in LPS-evoked neuroinflammatory responses and associated memory dysfunction.

## 2 Materials and methods

### 2.1 Behavioral study

Behavioral study was performed to investigate the effect of lupeol on memory functions by using a Morris water maze (MWM) task and a Y-maze task.

The experimental setup of MWM test comprised a circular water tank measuring 100 cm in diameter and 40 cm in height, containing opaque water at a temperature of 23 ± 1°C to a depth of 15.5 cm (22). Within this tank, a transparent escape platform measuring 10 cm in diameter and 20 cm in height was concealed 1 cm beneath the water’s surface, positioned at the midpoint of a designated quadrant. Each mouse underwent daily training sessions for five consecutive days, each involving a single hidden platform placed in one quadrant, with three rotating starting quadrants. The time taken by each mouse to escape from the water maze, locating the submerged escape platform, was recorded for every trial. On the fifth day, probe tests were conducted to assess memory consolidation. During the probe test, the platform was removed, and each mouse was allowed to swim freely for 60 s. The duration spent by the mice in the target quadrant, where the platform was situated was then measured. The time spent in the target quadrant is considered to represent the degree of memory consolidation that has taken place after learning. All data were recorded using video-tracking software (SMART, Panlab Harvard Apparatus Bioscience Company, United States).

The Y-maze apparatus consisted of three arms made up of transparent Plexiglas (23). Each arm measuring 50 cm in length, 20 cm in height, and 10 cm in width at both the bottom and top. Each mouse was placed at the center of the apparatus and allowed to move freely through the maze for three sessions of 8-min. Spontaneous alteration was defined as the successive entry of the mice into the three arms in overlapping triplet sets. Alteration behavior (%) was calculated as follows: [successive triplet sets (entries into three different arms consecutively)/total number of arm entries-2] × 100.

### 2.2 Animals grouping and treatment

**Figure 1:**
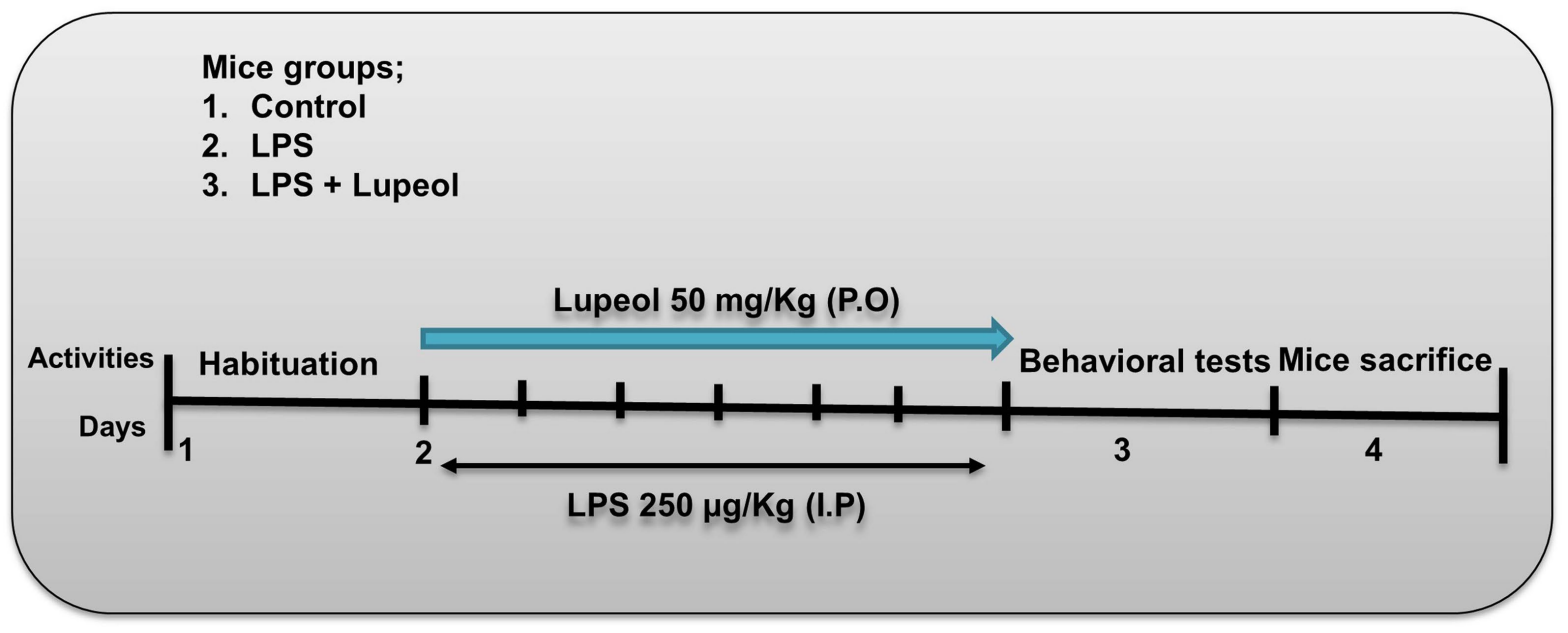
Experimental plan of lupeol against LPS-induced neuroinflammation mediated neurodegeneration in AD-Like pathologies.

Wild type C57BL/6 N male mice (*n* = 30, 8 weeks old, approximately 25-28 g body weight) were purchased from Samtako Biolabs (Ulsan, South Korea). All mice were housed in a temperature-controlled environment and maintained on a 12 h light/dark cycle with food *ad libitum*. Mice were randomly divided into 3 experimental groups as: Control group (0.9% saline I/P as control mice), LPS group (250 μg/kg I/P, for 7 days), LPS + Lupeol group (50 mg/kg orally, for 7 days) as mentioned in (Figure 1). LPS was dissolved in normal saline while lupeol was dissolved in an aqueous solution containing 0.25% DMSO. All the experimental procedures were carried out in accordance with the rules established by the animal ethics committee (IACUC) (approval ID: 125, animal ethics code: GNU-200331-M0020) of the Division of Applied Life Sciences, Department of Biology, Gyeongsang National University South Korea.

### 2.3 Protein extraction from mouse brain and assessment of acetylcholine activity

After behavioral analysis, using ketamine and xylazine the mice were anesthetized and sacrificed (24). The brain hippocampal tissues were dissected carefully and stored at −80°C. The hippocampus tissues were homogenized in PRO-PREP™ protein extraction solution (iNTRON Biotechnology, Inc., Sungnam, South Korea), and centrifuged at 13,000 rpm for 25 min at 4°C. The supernatants were collected and kept at −80°C for further process. Furthermore, the acetylcholinesterase (AChE) activities were also assessed in hippocampus of mice brain homogenates followed by Ellman protocol, which were standardized for the protein contents (5 mg/mL) (25–27).

### 2.4 Western blot analysis

Western blotting was performed as described in previous studies (28–30). Briefly a Bio-Rad assay kit (Bio-Rad Laboratories, Irvine, CA, United States) was used to measure protein concentrations. Consequently, proteins from the brain of all experimental groups of mice were run by SDS-PAGE on 4–18% gels in comparison under reducing conditions and subsequently transferred to polyvinylidene difluoride (PVDF) membranes (Immobilon-PSQ, Transfer membrane, Merck Millipore, Burlington, MA, United States). The membranes were blocked with 5% skim milk (Difco™ Skim Milk, BD, France), and incubated with primary antibodies at 4°C. After incubation, the membranes were probed with HRP-conjugated secondary antibodies. The detection was carried out using an enhanced chemiluminescent (ECL) reagent (ATTO Corporation, Tokyo, Japan), and the optical densities of the bands were quantified using ImageJ software.

### 2.5 Immunofluorescence assays

After anesthesia, transcardial perfusion was performed with normal saline (0.9%). The brains were carefully removed and preserved in ice-cold 4% neutral buffer paraformaldehyde at 4°C for 72 h. Subsequently, they underwent a dehydration process in 20% sucrose for another 72 h. Using a microtome (CM 3050C cryostat, Leica, Germany) on gelatin-coated slides, sections of 14 μm thickness were obtained (31). Immunofluorescence assays were conducted according to established protocols (32–34). Hippocampal tissue slides were then prepared, subjected to a 10 min wash with 0.01 M PBS, and treated with proteinase K for 5 min. Subsequent to PBS washing, sections underwent blocking with normal serum (Vector, diluted 1:20 in PBS) for 1 h. Incubation with specific antibodies (TNFα, IL-1β, Aβ, p-Tau, and PSD-95) was carried out overnight at 4°C. Following incubation, brain sections were washed with PBS and exposed to secondary antibodies that were tetramethylrhodamine isothiocyanate (TRITC) or fluorescein isothiocyanate (FITC) (anti-rabbit, anti-goat, or anti-mouse) diluted 1:50 in PBS for 90 min at room temperature. Tissue slides were then counterstained for 8 min with 4′,6-diamidino-2-phenylindole (DAPI) nucleus solution and further, the slides were prepared with mounting media by applying DPX (Distyrene Plasticizer Xylene), and protected with glass coverslips. Immunofluorescence imaging was performed using a confocal laser scanning microscope (FV 1000MPE, Olympus, Japan).

### 2.6 Antibodies

The primary antibodies in Table 1obtained from both Santa Cruz Biotechnology (Dallas, TX, United States) and Cell Signaling Technology.

**Table 1:** Antibodies used for immunofluorescence (IF) and Western Blot (WB) analysis.

Protein targets	Application/concentration	Catalog number	Manufacturer	Host
Anti-p-tau	WB/IF 1:1000/1:100	#12885	Cell Signaling Technology	Rabbit
Anti-APP	W.B 1:1000	#2452S	Cell Signaling Technology	Rabbit
Anti-Aβ	WB/IF 1:1000/1:100	SC-28365	Santa Cruz Biotechnology	Mouse
Anti-BACE1	WB 1:1000	SC-33711	Santa Cruz Biotechnology	Mouse
Anti-COX-2	WB 1:1000	SC-7951	Santa Cruz Biotechnology	Mouse
Anti-p-NfKB-p65	WB 1:1000	SC-136548	Santa Cruz Biotechnology	Mouse
Anti-TNF-α	WB/IF 1:1000/1:100	SC-52746	Santa Cruz Biotechnology	Mouse
Anti-IL-1β	WB/IF 1:1000/1:100	#12703	Cell Signaling Technology	Rabbit
Anti-PSD-95	WB/IF 1:1000/1:100	SC-71933	Santa Cruz Biotechnology	Mouse
Anti-SNAP-23	WB 1:1000	SC-374215	Santa Cruz Biotechnology	Mouse
Anti-SYP	WB 1:100	SC-17750	Santa Cruz Biotechnology	Mouse

### 2.7 Data analysis

Data were presented as mean ± standard error of the mean (SEM). Data were analyzed by ANOVA followed by a multi-comparison t-test. A level of (^#^*p* ≤ 0.01) and (^*^p ≤ 0.05) was considered to be significant. ‘#’ indicates a significant difference with the LPS-treated group, while ‘*’ indicates a significant difference with the control group.

## 3 Results

### 3.1 Lupeol inhibits LPS-induced elevated levels of p-NFκB and TNF-α

**Figure 2:**
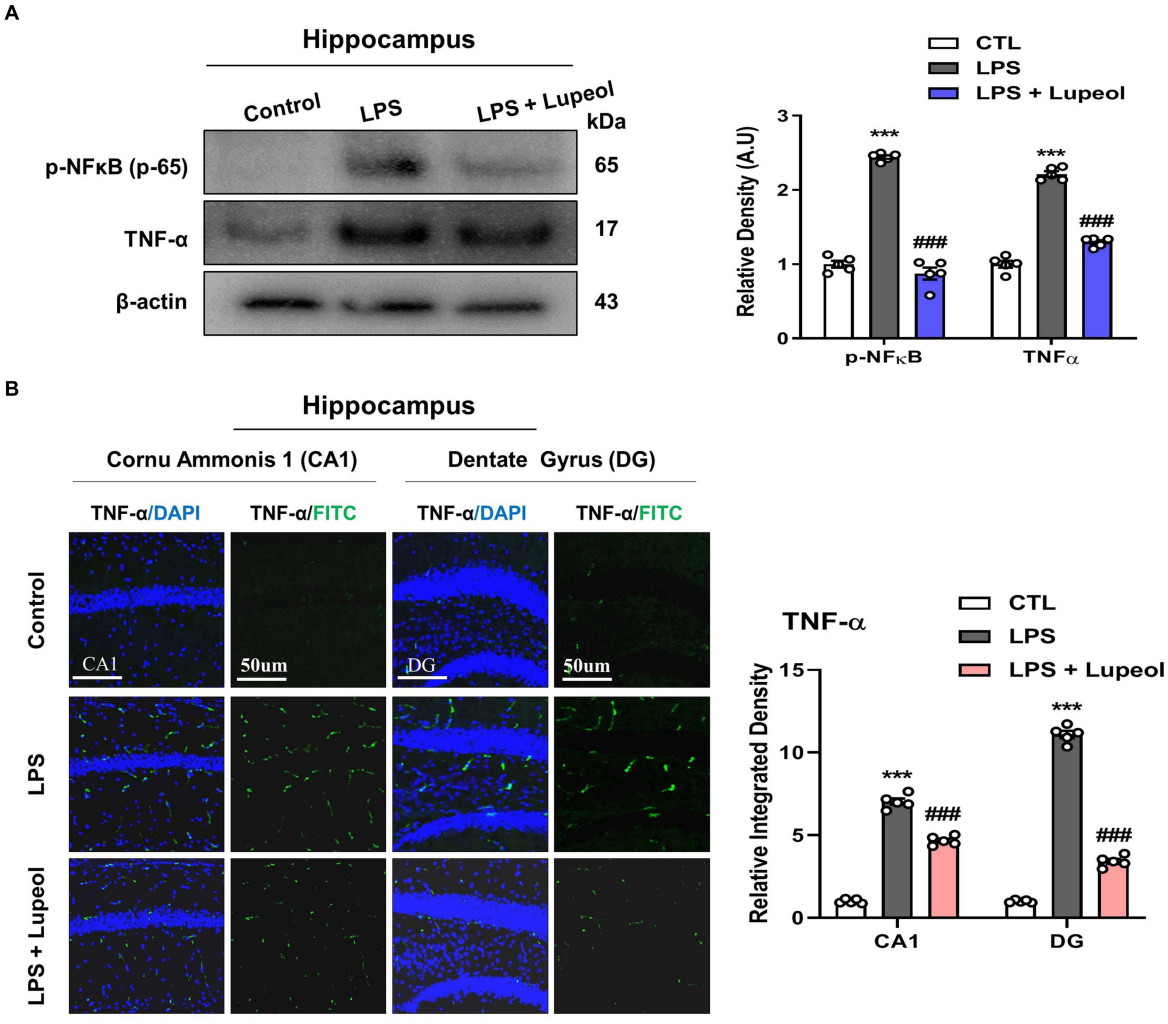
Lupeol inhibits LPS-induced elevated levels of p-NFκB and TNF-α. **(A)** Shown are representative western blots probed with antibodies of p-NFκB and TNF-α in the hippocampus of experimental mice. The protein bands were quantified using Sigma gel software. The density values are expressed in arbitrary units as the mean ± SEM for the indicated proteins (*n* = 5 animals per group). **(B)** Confocal microscopy represents immunofluorescence of TNF-α positive cells in the cornu ammonis 1 (CA1) and dentate gyrus (DG) regions of hippocampal mice brains. The symbol * showed a significant difference (*p ≤ 0.05) from control group, while symbol # represents a significant difference (^#^*p* ≤ 0.01) from the LPS-treated group.

To investigate the inhibitory effect of lupeol on memory impairment via preventing neuroinflammation, we determined the protein expressions of p-NFκB and TNF-α by western blot analysis. Our results showed that systemic administration of LPS significantly elevated the protein expression level of p-NFκB and TNF-α in the hippocampus of adult mice. Treatment with lupeol reversed the effect of LPS administration and reduced the elevated levels of p-NFκB and TNF-α compared to the LPS-treated group (Figure 2A). Likewise, immunofluorescence findings for TNF-α also showed that lupeol administration along with lupeol significantly inhibited the nuclear translocation of TNF-α compared to the LPS-induced effect (Figure 2B).

### 3.2 Lupeol inhibits LPS-induced elevated levels of inflammatory markers like COX-2 and IL-1β

**Figure 3:**
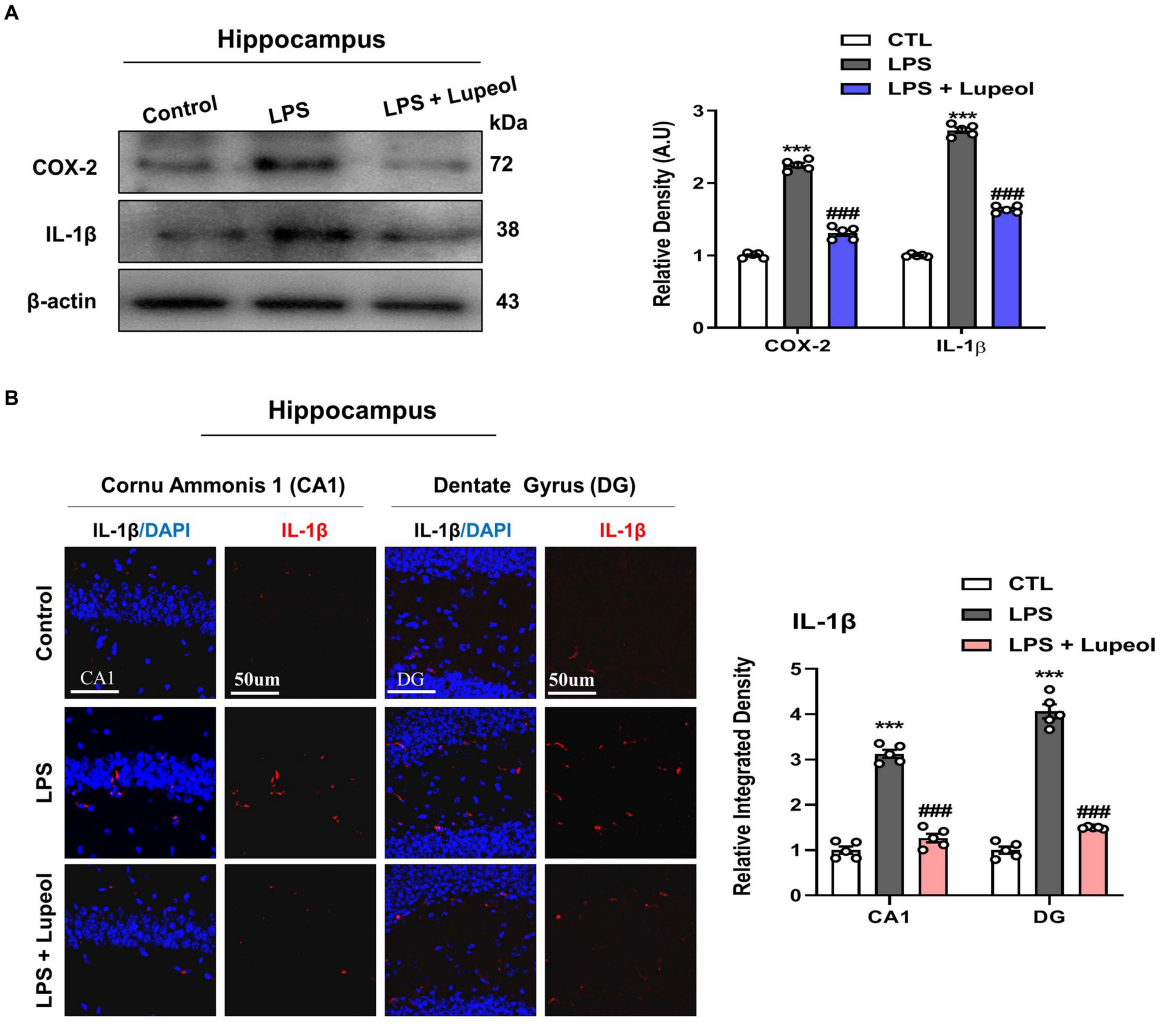
Lupeol inhibits LPS-induced elevated levels of COX-2 and IL-1β. **(A)** Shown are representative western blots probed with antibodies of COX-2 and IL-1β in the hippocampus of experimental mice. The protein bands were quantified using Sigma gel software. The density values are expressed in arbitrary units as the mean ± SEM for the indicated proteins (*n* = 5 animals per group). **(B)** Confocal microscopy shows immunoreactivity of IL-1β positive cells in the cornu ammonis 1 (CA1) and dentate gyrus (DG) regions of hippocampal mice brains. Symbol * showed a significant difference (^*^p ≤ 0.05) from control group, while symbol # represents a significant difference (^#^*p* ≤ 0.01) from the LPS-treated group.

We studied the effect of LPS and lupeol on proinflammatory mediators and cytokines such as COX-2 and IL-1β. Western blot analysis results showed that the protein expression level of these inflammatory markers was significantly elevated in the hippocampus of LPS-injected mice compared to the vehicle-treated mice. Systemic administration of lupeol lowered the LPS-induced increased expression of COX-2 and IL-1β showing a protective effect of lupeol in neuroinflammatory conditions (Figure 3A). Additionally, we performed immunofluorescence staining to determine the inhibitory effect of lupeol on the LPS induced expressions of inflammatory proteins. Our morphological results showed that LPS administration significantly elevated the expression level of IL-1β compared to the control group while lupeol treatment along with LPS showed a decreased hippocampal expression of IL-1β compared to the LPS-treated group (Figure 3B).

### 3.3 Lupeol inhibits LPS-induced elevated levels of amyloid precursor protein (APP) and Aβ expressions

**Figure 4:**
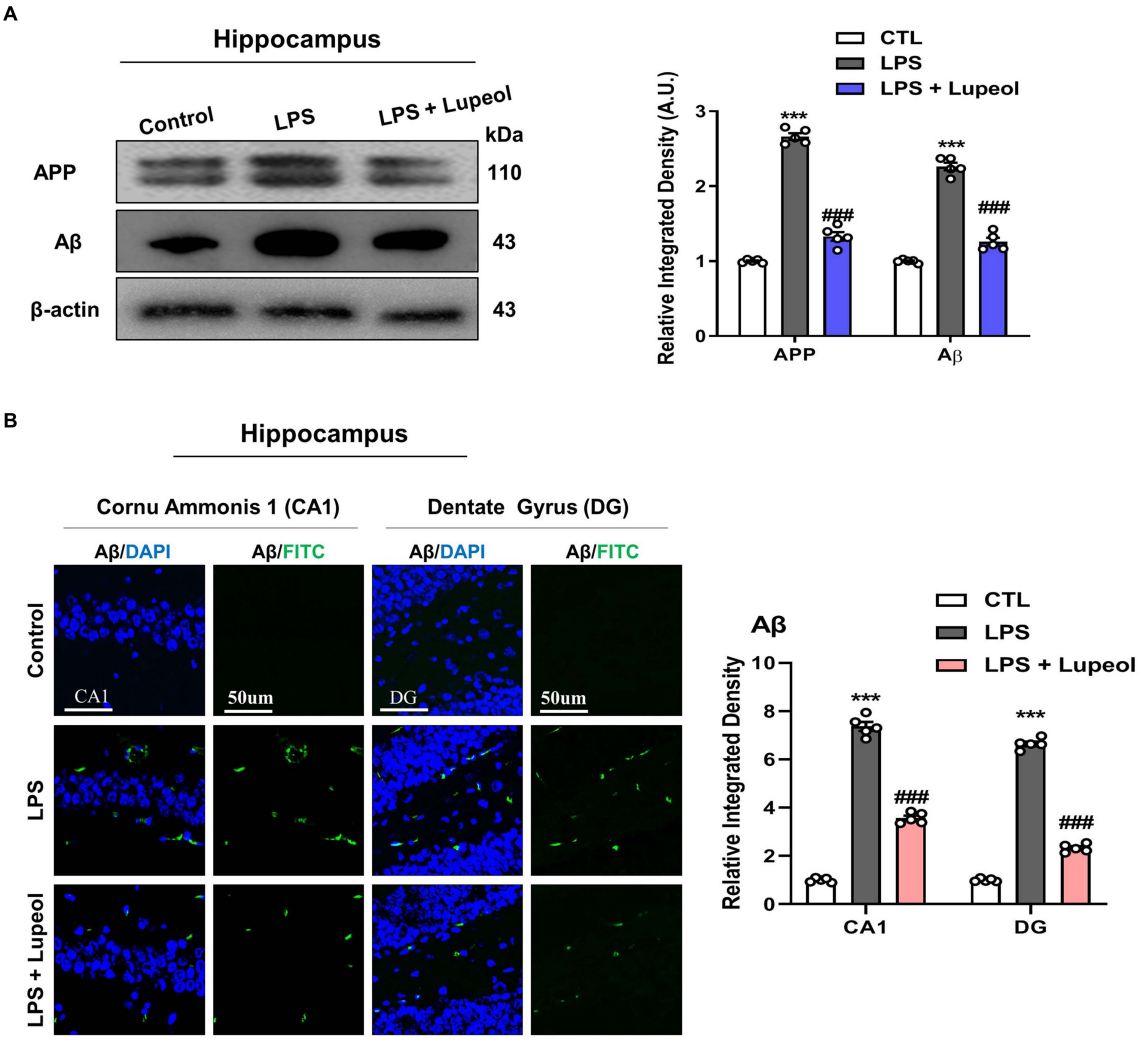
Lupeol inhibits LPS-induced elevated levels of APP and Aβ. **(A)** Shown are representative western blots probed with antibodies of APP and Aβ in the hippocampus of experimental mice. The protein bands were quantified using Sigma gel software. The density values are expressed in arbitrary units as the mean ± SEM for the indicated proteins (*n* = 5 animals per group). **(B)** Shown are representative immunofluorescence photomicrographs of Aβ positive cells in the cornu ammonis 1 (CA1) and dentate gyrus (DG) regions of hippocampal mice brains. Symbol * showed a significant difference (^*^p ≤ 0.05) from control group, while symbol # represents significant difference (^#^*p* ≤ 0.01) from the LPS-treated group.

Next, to determine the effect of the administration of LPS and lupeol on Alzheimer-associated proteins like APP and Aβ, Western blot analysis was performed. LPS treatment of 250ug/kg for 1 week induced an Alzheimer-like effect and showed an increased protein expression level of APP and Aβ in the hippocampus as compared to the vehicle-treated group. Treatment with lupeol attenuated the LPS-induced elevated level of APP and Aβ as compared to the LPS-treated group (Figure 4A). Similarly, confocal microscopy results showed that lupeol administration significantly inhibited the LPS-induced elevated expression of Aβ as compared to the LPS-treated group (Figure 4B).

### 3.4 Lupeol inhibits LPS-induced elevated levels of BACE-1 and p-tau

We also investigated the effect of LPS and lupeol on other Alzheimer-associated proteins like BACE-1 and hyperphosphorylated tau. Tau is a neuronal microtubule-associated protein and its major biological function is to maintain microtubule assembly. The abnormal hyperphosphorylation of tau (p-Tau) is responsible for the loss of normal physiological functions and is mainly involved in the pathophysiology of AD (35, 36).

**Figure 5:**
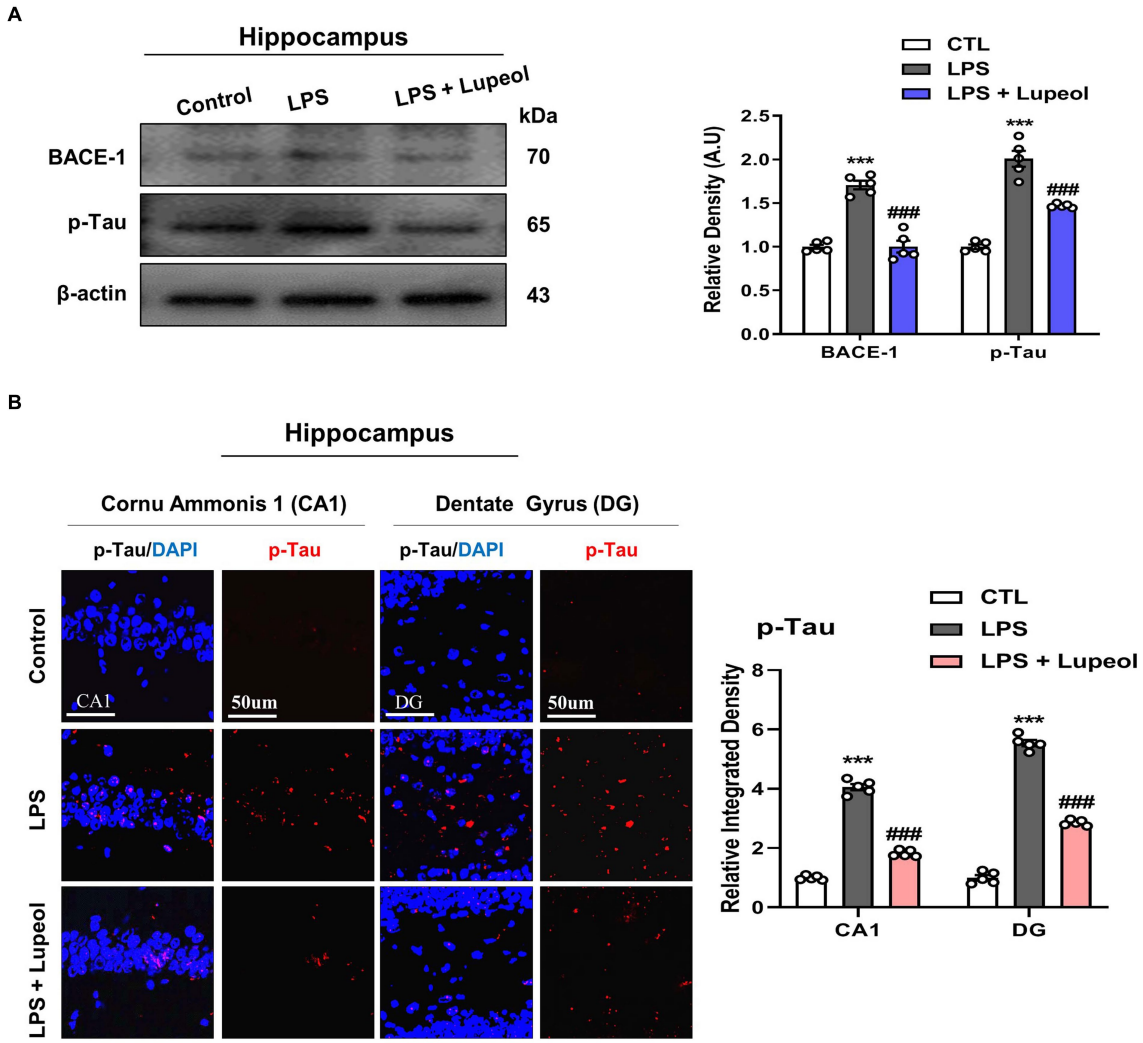
Lupeol inhibits LPS-induced elevated levels of BACE-1 and p-Tau**. (A)** Shown are representative western blots probed with antibodies of BACE-1 and p-Tau in the hippocampus of experimental mice. The protein bands were quantified using Sigma gel software. The density values are expressed in arbitrary units as the mean ± SEM for the indicated proteins (*n* = 5 animals per group). **(B)** Shown are representative immunofluorescence photomicrographs of p-Tau positive cells in the cornu ammonis 1 (CA1) and dentate gyrus (DG) regions of hippocampal mice brains. Symbol * showed a significant difference (^*^p ≤ 0.05) from the control group, while symbol # represents significant difference (^#^*p* ≤ 0.01) from the LPS-treated group.

Western blot analysis was performed and our result showed that systemic administration of LPS significantly elevated the protein expression level of BACE-1 and p-Tau in the hippocampus. Oral administration of lupeol along with LPS significantly decreased the expression level of BACE-1 and p-Tau as compared to LPS-treated (Figure 5A). Furthermore, we also performed immunofluorescence staining for p-Tau in the hippocampus of the experimental groups. The results were consistent with the Western blot result as lupeol significantly reduced the LPS-induced hyperphosphorylation of Tau protein as compared to the LPS-treated group (Figure 5B).

### 3.5 Lupeol inhibits LPS-induced elevated levels of PSD-95, SNAP-23, and SYP

A number of research studies have shown that disruption of synaptic function is one of the main features of AD, which may result in cognitive dysfunction and memory impairment (37, 38).

**Figure 6:**
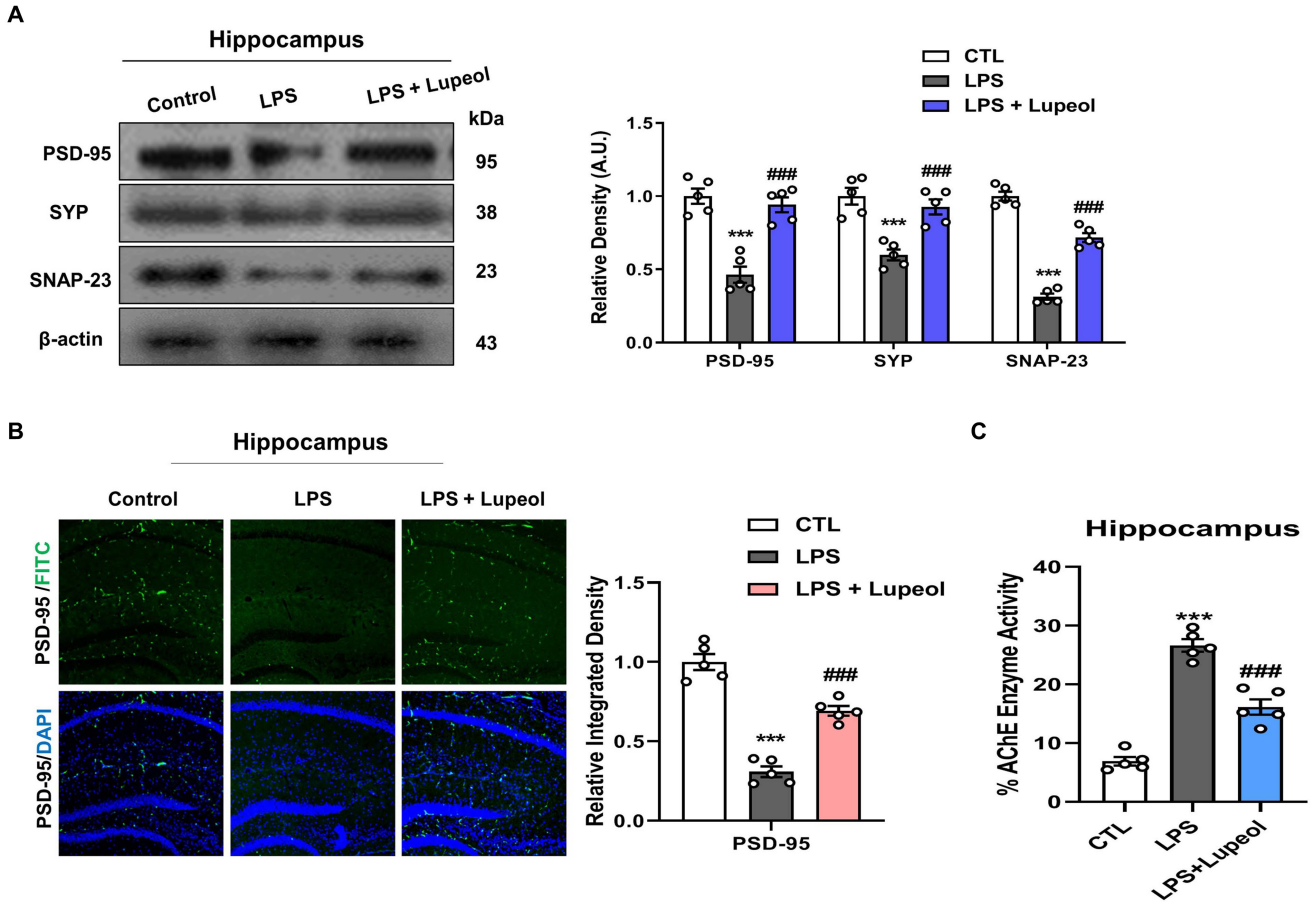
Lupeol inhibits LPS-induced elevated levels of PSD-95, SYP, and SNAP-23. **(A)** Shown are representative western blots probed with antibodies of PSD-95, SYP, and SNAP-23 in the dentate gyrus (DG) of hippocampus of experimental mice. The protein bands were quantified using Sigma gel software. The density values are expressed in arbitrary units as the mean ± SEM for the indicated proteins (*n* = 5 animals per group). **(B)** Shown are representative immunoreactivity of PSD-95 positive cells in the hippocampal mice brain. **(C)** Indicates Acetylcholinesterase (AChE) in mouse brain hippocampal tissue. Symbol * showed a significant difference (^*^p ≤ 0.05) from control group, while symbol # represents a significant difference (^#^*p* ≤ 0.01) from LPS-treated group.

In order to investigate the change in synaptic function by treatment with LPS and lupeol, western blot analyses were performed. The protein expression level of pre- and post-synaptic markers like PSD-95, SNAP-23, and SYP were determined. Our results showed that systemic administration of LPS significantly decreased the protein expression level of pre-synaptic markers like SNAP-23 and SYP, and post-synaptic markers like PSD-95 in the hippocampus, while administration of lupeol reverses the effect of LPS and increased expression of PSD-95, SNAP-23, and SYP (Figure 6A). In accordance with these, morphological results for PSD-95 also showed a significant decrease in immunofluorescence results of PSD-95 in LPS treated group as compared to vehicle-treated group. Treatment with lupeol showed an increased expression of PSD-95 in the hippocampus as compared to the LPS-treated group (Figure 6B).

### 3.6 Lupeol reduced acetylcholinesterase activity in LPS-induced neuroinflammation in Alzheimer’s disease associated pathology

Acetylcholinesterase (AChE) plays a critical role in the hydrolysis of acetylcholine, a neurotransmitter essential for cognitive function. The study demonstrated that LPS-induced neuroinflammation significantly increased AChE activity in the hippocampus of LPS treated group of animals, mimicked AD-like cholinergic dysfunction. While lupeol administration at 50 mg/kg dose effectively reduced AChE activity as compared to LPS treated adult mice showing a more pronounced effect. These findings suggest that lupeol mitigates cholinergic deficits associated with neuroinflammation and highlights its potential as a therapeutic agent for Alzheimer’s disease (Figure 6C) (39, 40).

### 3.7 Lupeol ameliorated cognitive functions in LPS-administered mice

Natural-derived compounds such as flavonoids show great potential in improving learning and memory functions has been confirmed by various research studies (5, 41, 42). Lupeol also has a beneficial effect on memory and cognitive functions (43, 44). Nevertheless, several studies have explored that systemic LPS administration induces memory and cognitive dysfunction (45–47). Consequently, to evaluate the memory-improving effect of lupeol against LPS, we designed a dosage treatment of lupeol at a 50 mg/kg body weight dose for one week (cotreated with LPS) via orally. Other studies also mentioned lupeol of 50 mg/kg /day P.O. for a short period of time induce beneficial effects (21, 43).

**Figure 7:**
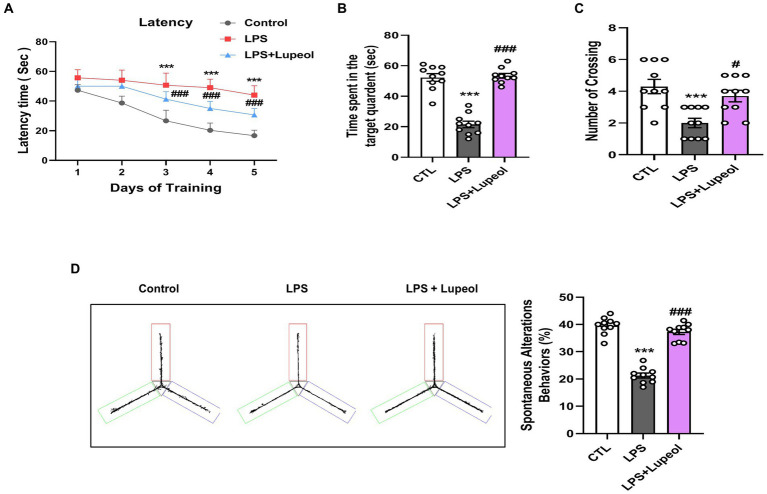
Lupeol enhanced memory function in mice treated with LPS. Behavioral assessments were conducted using the MWM and Y-maze tests to assess memory function in control mice, LPS and LPS + lupeol group mice. **(A)** Average escape latency time for experimental mice to reach the hidden platform from 1 to 4 days. **(B)** Time spent in the platform quadrant, where the hidden platform was placed during the trial session. **(C)** The average number of crossings at the hidden platform during the probe test of the MWM test. **(D)** Spontaneous alteration behavior % of the mice during the Y-maze test. Histograms indicate the means ± SEM for the mice (*n* = 15/group). ^∗^Significantly different from the control; # significantly different from LPS-treated group. Significance: ^∗^*p* ≤ 0.05, ^#^*p* ≤ 0.01.

We assessed the memory capabilities of the mice through MWM and Y-maze assessments. In the MWM task, all mice underwent training to locate a submerged platform, after which we analyzed the time taken to reach it. The mice injected with LPS exhibited prolonged search times compared to the control group (Figure 7A). Nonetheless, administration of lupeol countered the impact of LPS, notably enhancing memory performance, as evidenced by the reduced duration required by the subjects to locate the concealed platform in comparison to mice injected only with LPS. Additionally, the probe test revealed that lupeol also counteracted the effects of LPS, resulting in a significant increase in the number of crosses across the platform and an increase in the duration spent within the specific quadrant where the concealed platform had previously been positioned (Figures 7A–C). These results revealed that lupeol reversed the harmful effect of LPS and significantly enhanced memory functions. The findings from the Y-maze experiment show that compared to the control group, LPS caused short-term spatial memory impairment. However, treatment with lupeol significantly increased the percentage of spontaneous alteration behavior, suggesting an improvement in spatial working memory function in LPS-injected mice (Figure 7D).

## 4 Discussion

Although extensive ongoing research is investigating various therapeutic agents for treating neurodegenerative diseases such as Alzheimer’s disease (AD), no single intervention has been found to comprehensively address the treatment of AD. It has been widely studied that neuroinflammation plays a crucial role in the progression of neurological disorders especially AD (48–50). Several lines of evidence supported that neuroinflammatory mechanisms are involved in the pathogenesis of neurodegenerative disorders like AD. Induction of inflammatory conditions by an exogenous stimulant like LPS is well studied and it has been described that LPS administration alleviates the level of pro-inflammatory mediators and other cytokines (51, 52). The release of these inflammatory cytokines like TNF-α, IL-1β, NOS-2, and prostaglandins are the intermediary mediators to induce neuronal injury and apoptosis (53, 54). In this regard, therapeutic agents having potential anti-inflammatory activity can be considered suitable candidates to target inflammation-induced neurological disorders. In the present study, we described that the anti-inflammatory activity of lupeol may be attributed to memory improvement in a mouse model of Alzheimer’s disease.

Previously, lupeol has been extensively studied both *in vitro* and *in vivo* for its anti-inflammatory and anti-carcinogenic properties (55, 56). Furthermore, we showed that lupeol inhibits LPS-induced neuroinflammation and neurodegeneration via preventing the p38/JNK pathway (57). It has been reported that both P38/JNK pathway and NFκB pathway are major inflammatory pathways involved in neuroinflammation-induced neurodegeneration (58, 59).

It is determined that LPS administration in adult mice develops neuroinflammatory conditions with enhanced release of inflammatory cytokines that ultimately leads to memory impairment and cognitive dysfunction (60, 61). Geetha et al. (56) checked the anti-inflammatory activities of lupeol in rat arthritis model, so we hypothesize that whether lupeol could reduce the inflammation in the brain. In our previous study, we showed that neuroinflammatory markers like TNF-alpha, NOS2, and IL-1β were decreased in lupeol treated LPS model. This finding was validated in our current study and further investigated neuroinflammation markers such as NF-ĸB and COX2. Also, our previous study did not examine the amyloid beta-related pathology and pre- and post-synaptic markers. Therefore, the findings from this study further increased our knowledge in the neuroprotective properties of lupeol. Likewise, other studies have described disrupted synaptic and memory function involving increased levels of Alzheimer’s markers like APP and Aβ, BACE-1, and p-Tau with LPS administration in rodents (62–64). It has been demonstrated that NFκB regulates Aβ production as the promoter region of β-secretase (BACE-1) has NFκB binding site, also stated that inflammatory cytokine-like TNF-α and IFN in combination provokes the production of Aβ peptides (65, 66). Furthermore, it has been well-studied in human and animal models that neuroinflammation, amyloid accumulation, and other phenomena of AD lead to local synaptic damage and memory deficits (67). A number of research bodies have shown that pre-synaptic markers like SYP, SNAP-25, SNAP-23, etc., and post-synaptic markers like PSD-95 are associated with synaptic plasticity and cognitive function (68). It has been demonstrated that the expression of pre-and post-synaptic markers has been reduced to a significant level in AD patients as well as in rodent’s animal models (69, 70). In line with these studies, our results showed that LPS treatment enhanced the level of inflammatory markers like NFκB, TNF-α, and IL-1β, and Alzheimer’s associated proteins like APP and Aβ, and p-Tau while lupeol administration significantly inhibited the LPS-induced effect on neuroinflammatory markers, AD markers and pre-synaptic markers like SNAP-23 and SYP, and post-synaptic markers like PSD-95. Moreover, lupeol significantly reduced AChE activity in LPS-induced neuroinflammation model of Alzheimer’s disease like pathologies. These results provide the neuroprotective role of lupeol and its therapeutic potential in regulating cholinergic dysfunctions in AD (71, 72).

In conclusion, our results showed that lupeol possess properties to inhibit LPS-induced expression of pro-inflammatory mediators, which may further lead to inhibition of inflammation-induced memory impairment and synaptic dysfunction. However, we suggest that detail experimental research need to be performed for considering it a potential treatment for neuroinflammatory diseases and inflammation-induced neurological disorders like AD.

## Data availability statement

The raw data supporting the conclusions of this article will be made available by the authors, without undue reservation.

## Ethics statement

The animal study was approved by Animal Ethics Committee (IACUC) of the Division of Applied Life Science, Gyeongsang National University, Jinju, South Korea. The study was conducted in accordance with the local legislation and institutional requirements.

## Author contributions

KC: Conceptualization, Data curation, Formal analysis, Investigation, Methodology, Software, Writing – original draft, Writing – review & editing. JSP: Conceptualization, Data curation, Formal analysis, Investigation, Methodology, Software, Writing – original draft, Writing – review & editing. HYP: Conceptualization, Data curation, Formal analysis, Investigation, Methodology, Software, Writing – original draft, Writing – review & editing. MT: Formal analysis, Methodology, Writing – review & editing. TJP: Formal analysis, Investigation, Supervision, Writing – review & editing. MOK: Formal analysis, Investigation, Supervision, Writing – review & editing.
